# Research on Mechanical Properties and Crack Evolution of Basalt Fiber-Reinforced Coal Gangue–Slag Geopolymer Concrete Based on Digital Image Correlation

**DOI:** 10.3390/ma19101995

**Published:** 2026-05-12

**Authors:** Weizi Wang, Lianyong Zhu, Jingcheng Ju, Xiaotong Gao, Xi Chen

**Affiliations:** 1 School of Water Conservancy and Architectural Engineering, Tarim University, Alar 843300, China; wangweizi1224@163.com (W.W.); 18731380560@163.com (X.G.); 2 Qingdao Fangyuanda Construction Group Co., Ltd., Qingdao 266000, China; jujc1227@163.com

**Keywords:** basalt fiber, coal gangue–slag geopolymer concrete, digital image correlation, crack evolution, mechanical properties

## Abstract

To investigate the influence of basalt fiber (BF) on the mechanical properties and crack evolution of coal gangue–slag geopolymer concrete, geopolymer concrete specimens were prepared using coal gangue powder calcined at 700 °C and slag as precursors, with BF contents ranging from 0 to 1.25%. Mechanical testing combined with digital image correlation (DIC), scanning electron microscopy (SEM), and X-ray diffraction (XRD) was conducted to evaluate the effects of BF on macroscopic mechanical behavior, crack evolution, and underlying microstructural mechanisms. The results demonstrate that BF effectively enhances both the mechanical performance and crack-control capacity of coal gangue–slag geopolymer concrete, exhibiting a clear content-dependent trend. Compressive strength initially increases and subsequently decreases with increasing BF content. The 28-day compressive strength reaches a maximum value of 84.05 MPa at a BF content of 0.5%, representing an 11.92% improvement compared with the control group. Splitting tensile strength and flexural strength attain their peak values at a BF content of 1%, increasing by 37.88% and 25.81%, respectively. DIC analysis indicates that BF delays strain localization and effectively restrains the propagation of dominant cracks. Specifically, the compressive strain field becomes more uniformly distributed at 0.5% BF content, while crack propagation during splitting failure is more stable at 1% BF content. SEM observations reveal that the primary strengthening mechanisms include crack bridging, interfacial load transfer, and energy dissipation associated with fiber pull-out. XRD analysis shows that BF incorporation does not significantly alter the phase composition of the coal gangue–slag geopolymer system; thus, performance enhancement mainly arises from fiber bridging and interfacial reinforcement rather than changes in primary reaction products.

## 1. Introduction

The production of ordinary Portland cement is associated with high energy consumption and significant CO_2_ emissions, making the development of low-carbon cementitious materials an important research direction in the field of construction materials [1,2,3]. Concrete is among the most extensively used construction materials worldwide, and Portland cement is the major contributor to carbon emissions associated with concrete production [4,5,6]. Therefore, the development of low-carbon cementitious systems capable of replacing traditional cement is of considerable practical and environmental significance.

Geopolymer concrete (GPC) generally refers to a cement-free or low-cement material in which ordinary Portland cement is replaced by a binder formed through the alkali activation of aluminosilicate precursors, combined with coarse and fine aggregates [7,8,9,10]. Common precursors include fly ash, slag, and metakaolin. Coal gangue is a bulk solid waste generated during coal mining and washing, primarily composed of SiO_2_ and Al_2_O_3_, with mineral phases dominated by kaolinite and quartz [11,12]. Raw coal gangue exhibits low reactivity; however, calcination induces dehydroxylation of kaolinite, transforming it into a highly reactive aluminosilicate phase similar to metakaolin, thereby significantly enhancing its pozzolanic activity and chemical reactivity [13]. Consequently, calcined coal gangue can serve as an effective active precursor in geopolymer systems for concrete production [14].

Despite these advantages, GPC remains an inherently quasi-brittle material. Once cracks initiate, they propagate rapidly, and the post-peak load-bearing capacity decreases sharply, resulting in pronounced brittle failure characteristics [15,16]. Therefore, understanding fracture behavior and improving brittleness resistance are critical for ensuring structural safety and service reliability. In recent years, fiber-reinforced geopolymer concrete (FRGPC) has emerged as an effective approach to mitigating the brittleness of GPC through the incorporation of discrete reinforcing materials such as steel fibers, basalt fibers, and polypropylene fibers into the geopolymer matrix [17,18,19,20]. Compared with unreinforced GPC, FRGPC typically exhibits higher tensile and flexural strength, improved fracture toughness and energy dissipation capacity, and enhanced crack control and deformation compatibility [21,22,23,24,25,26]. Among these reinforcing materials, basalt fiber has attracted increasing attention because of its high tensile strength, high elastic modulus, low density, good corrosion and alkali resistance, thermal stability, and favorable compatibility with inorganic matrices. Existing studies indicate that basalt-fiber reinforcement can be introduced not only in the form of short/chopped fibers, but also as continuous or unidirectional composite reinforcements. Saranya et al. reported that steel fibers significantly improve the engineering performance and durability of GGBS–dolomite geopolymer concrete while enhancing bond strength and impact resistance [21]. Şahin et al. demonstrated the beneficial effects of basalt fibers on metakaolin-based geopolymer mortar [22]. Li et al. showed that polypropylene fibers effectively improve flexural fracture parameters and resistance to crack propagation in geopolymers [23]. Su et al. found that steel fibers increase the static compressive strength of geopolymer concrete by 23.2% and enhance dynamic compressive performance and energy absorption capacity [24]. From a fracture-mechanics perspective, Gomes et al. and Wang et al. further confirmed that steel and basalt fibers reduce brittle failure characteristics by bridging cracks, delaying crack propagation, and increasing fracture energy [25,26]. More recently, Furtos et al. showed that continuous MiniBars^TM^ basalt fiber reinforcement can significantly improve the flexural and tensile performance of geopolymer composites, highlighting the effectiveness of basalt-based reinforcement beyond conventional short-fiber forms [27]. Zhang et al. further reported that basalt fiber-reinforced geopolymer concrete exhibited improved long-term durability in marine environments, while Wang et al. showed that basalt fiber-reinforced geopolymer repair materials can enhance interfacial bond stability under high-temperature conditions [28,29]. In general, basalt fibers are particularly effective in improving tensile, flexural, fracture-related, and crack-control behavior through crack bridging, interfacial load transfer, and pull-out-related energy dissipation.

Digital image correlation (DIC) technology enables continuous monitoring of crack initiation, propagation, and opening evolution and has been widely applied in studies of fracture behavior in concrete and geopolymer materials [26,30,31]. Current research on coal gangue–slag geopolymer concrete primarily focuses on mechanical properties and durability, whereas studies addressing fracture evolution and strain localization processes remain limited [32,33,34]. Ma et al. reported that coal gangue–slag alkali-activated concrete exhibits high compressive strength and good durability, and that calcined coal gangue coarse aggregates further enhance sulfate resistance [32]. Zhu et al. demonstrated excellent early compressive strength and chloride ion penetration resistance in alkali-activated coal gangue–slag concrete and established cumulative damage models under freeze–thaw and salt-freezing conditions [33]. Zhang et al. showed that appropriate slag incorporation systematically improves mechanical properties, frost resistance, and chloride penetration resistance, with durability closely related to pore-structure optimization and reaction-product evolution [34].

Existing studies indicate that basalt fibers can enhance macroscopic mechanical properties, fracture toughness, and deformation behavior in geopolymer and concrete systems, while DIC has been successfully employed to characterize fiber-regulated crack propagation processes [35,36,37]. Li et al. found that basalt fibers improve the mechanical performance and sulfate erosion resistance of alkali-activated fly ash–slag coal gangue pervious concrete [35]. Yang et al. found that the incorporation of basalt fibers improved the compressive, flexural, and splitting tensile properties of coal gangue coarse aggregate–fly ash geopolymer concrete and also enhanced its stress–strain response and deformation failure characteristics [36]. Lian et al. demonstrated that basalt fibers significantly enhance the fracture performance of nano-CaCO_3_ concrete, with optimal improvement observed at a fiber content of 0.2%, and DIC effectively captured the full crack propagation process [37]. An et al. further showed through full-field strain analysis that basalt fibers modify crack evolution under compression, promoting dispersed microcrack development and increasing the complexity of macroscopic crack paths [38]. Nevertheless, systematic investigations remain limited regarding the coupled relationship among strain localization, main crack propagation, and microscopic interfacial mechanisms throughout the compressive and splitting tensile processes of coal gangue–slag composite precursor concrete.

Nevertheless, systematic investigations remain limited regarding the coupled relationship among mechanical performance, crack evolution, and microscopic mechanisms in basalt fiber–reinforced coal gangue–slag geopolymer concrete. To address this issue, the present study employs calcined coal gangue powder and slag as composite precursors, with basalt fiber content selected as the primary variable. The novelty of this work lies not only in investigating a relatively less explored basalt fiber–reinforced coal gangue–slag geopolymer concrete system, but also in establishing a multiscale analytical framework linking macroscopic mechanical behavior, full-field strain evolution, crack propagation, and microscopic mechanisms. Unlike previous studies that mainly focused on conventional geopolymer systems or only reported strength enhancement, this study combines compressive, splitting tensile, and flexural tests with DIC, SEM, and XRD analyses to systematically evaluate the effects of basalt fibers on coal gangue–slag geopolymer concrete. Specifically, DIC is used to reveal strain localization, crack initiation, and propagation behavior during compressive and splitting tensile loading; SEM is applied to characterize toughening mechanisms such as fiber bridging, interfacial bonding, and pull-out energy dissipation; and XRD is employed to examine whether fiber incorporation alters the main phase composition of the geopolymer matrix. By correlating macroscopic performance with crack evolution and microscopic observations, this study further clarifies the strengthening and crack-resistance mechanisms induced by basalt fibers and reveals that the optimal fiber content depends on the loading mode, with distinct optimal dosages for compression-dominated and tension-dominated performance. These findings provide a scientific basis for the optimized design and engineering application of solid-waste-based geopolymer concrete.

## 2. Experiment

### 2.1. Experimental Materials

The coal gangue–slag geopolymer concrete (CGGPC) investigated in this study was composed of calcined coal gangue powder, ground granulated blast furnace slag (GGBFS), fine aggregate, coarse aggregate, an alkali activator, and basalt fibers. The cementitious binder adopted a composite system consisting of calcined coal gangue powder and S95-grade GGBFS. The calcined coal gangue powder was supplied by Yunshi Calcined Coal Gangue Powder Mineral Products Processing Plant (Lingshou County, Hebei Province, China). It appeared as a gray powder with an average particle size of approximately 18 μm and a density of about 2.8 g/cm^3^. The slag, provided by Hebei Jiegui Mineral Products Co., Ltd. (Shijiazhuang, China), was a white powder with an average particle size of approximately 75 μm and a density of 3.12 g/cm^3^. The main chemical compositions of the two precursors are listed in Table 1. Test results indicate that the slag contains 35% CaO, 33.5% SiO_2_, and 17.5% Al_2_O_3_, while the coal gangue contains 53.5% SiO_2_ and 42% Al_2_O_3_. The combination of these materials provides sufficient silica and alumina sources together with an appropriate calcium supply to facilitate geopolymerization reactions.

The alkali activator was prepared using sodium silicate solution, flake sodium hydroxide (NaOH), and mixing water. The initial modulus of sodium silicate was 3.21, which was adjusted to 1.3 by adding flake NaOH with a purity of 96%. Natural river sand was used as the fine aggregate, with a fineness modulus of 2.54 and an apparent density of 2635 kg/m^3^. Crushed stone with particle sizes of 5–10 mm and 10–20 mm served as the coarse aggregate, and the two size fractions were blended at a mass ratio of 5:5. Basalt fiber (BF) was employed as the reinforcing and toughening material. An alkali-resistant basalt fiber was used in this study. The physical and mechanical properties listed in Table 2 were obtained from the manufacturer’s technical datasheet. The fibers had a length of 12 mm, a density of 2.69 g/cm^3^, a tensile strength exceeding 2000 MPa, an elastic modulus greater than 85 GPa, a diameter of approximately 17 μm, and an elongation at break of 2.5%. These properties indicate high strength and stiffness, making basalt fiber suitable for enhancing crack resistance and toughness in geopolymer concrete. The detailed fiber parameters are presented in Table 2.

### 2.2. Specimen Design and Mix Proportion

Based on preliminary experiments and relevant literature [39,40,41], the alkali activator modulus was fixed at 1.3 and the alkali equivalent at 9%. The water-to-binder ratio, aggregate-to-binder ratio, and sand ratio were set at 0.41, 3.5, and 0.37, respectively. A composite binder with a mass ratio of calcined coal gangue powder to slag of 1:1 was adopted. Basalt fiber content was selected as the sole experimental variable, with volume fractions of 0, 0.25%, 0.50%, 0.75%, 1.00%, and 1.25% relative to the total volume of geopolymer concrete. The selected range was based on preliminary experiments and previous studies [35,36]. Accordingly, six mixtures were designed, namely JZ, BF0.25, BF0.5, BF0.75, BF1, and BF1.25. The quantities of calcined coal gangue powder, slag, water glass, NaOH, water, sand, coarse aggregate, and basalt fiber for each mixture were determined on a unit-volume basis (kg/m^3^), as summarized in Table 3. It was intended to cover both low-fiber content, where the reinforcing effect may be limited, and relatively high-fiber content, where workability loss and fiber agglomeration may occur. The detailed mix proportions are listed in Table 3.

### 2.3. Preparation of Specimens

**Figure 1:**
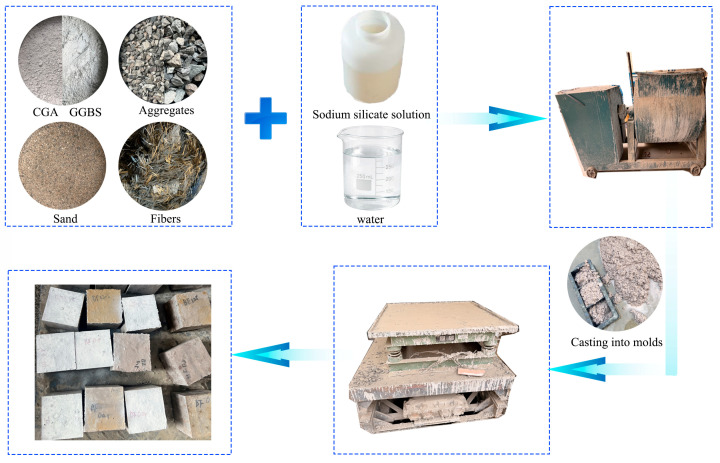
Preparation flow chart of specimens. The arrows indicate the sequence of specimen preparation.

The specimen preparation procedure is illustrated in Figure 1. The alkali activator was prepared 12 h prior to mixing. Pre-weighed sodium silicate solution was placed in a container, and solid NaOH was gradually added under continuous stirring until completely dissolved to ensure the designed activator modulus. Subsequently, calcined coal gangue powder, slag, fine aggregate, and coarse aggregate were weighed according to the mix design and dry-mixed for 3 min to achieve uniform distribution of solid constituents. Basalt fibers were incorporated in two stages during mixing. Approximately half of the fibers were slowly dispersed into the dry mixture at the beginning of mixing to promote uniform dispersion. The prepared alkali activator solution was then added gradually, followed by an additional 3 min of mixing, during which the remaining fibers were evenly introduced to minimize fiber clustering and local agglomeration. After mixing, the fresh concrete was poured into previously prepared molds and consolidated on a vibration table. The specimens were removed from the molds after 24 h and then cured under standard conditions at (20 ± 2) °C with a relative humidity above 95% until the designated testing age.

### 2.4. Test Methods

#### 2.4.1. Strength Tests

**Figure 2:**
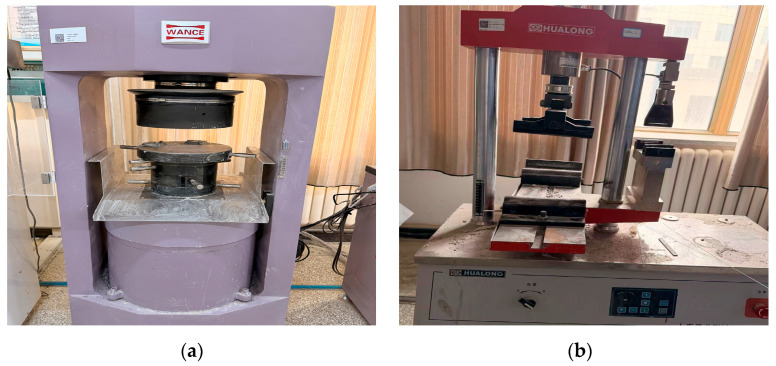
Mechanical property testing equipment: (**a**) computer-controlled fully automatic pressure testing machine; (**b**) computer-controlled electronic flexural testing machine.

According to the provisions of the Standard Test Methods for Physical and Mechanical Properties of Concrete (GB/T 50081-2019) [42], a computer-controlled automatic compression testing machine (HCT306A, accuracy ±1%, Shenzhen WANCE Testing Equipment Co., Ltd., Shenzhen, China) was used for the compressive and splitting tensile tests, whereas a computer-controlled electronic flexural testing machine (WHY-300, Shanghai Hualong Testing Instruments Co., Ltd., Shanghai, China) was adopted for the flexural tests, as shown in Figure 2. Three parallel specimens were tested for each group, and the average value was taken as the representative result. Cubic specimens of 100 mm × 100 mm × 100 mm were used for compressive and splitting tensile tests, whereas prism specimens of 100 mm × 100 mm × 400 mm were used for flexural tests.

#### 2.4.2. Digital Image Correlation Test

**Figure 3:**
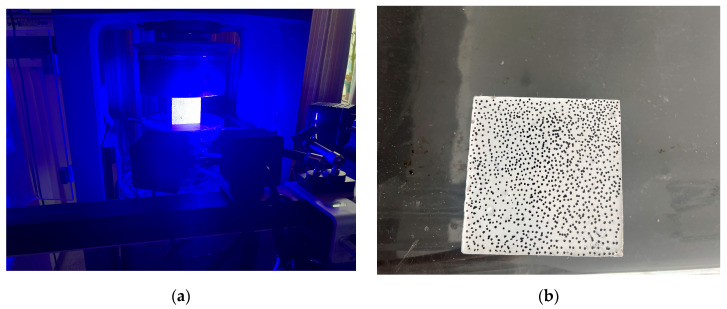
Digital image correlation (DIC) testing system and speckle pattern on specimen surface: (**a**) DIC testing device; (**b**) speckle pattern on specimen surface.

To investigate deformation localization and crack evolution during compressive and splitting tensile loading, digital image correlation (DIC) technology was employed for non-contact full-field displacement and strain measurements, as shown in Figure 3a. A two-dimensional DIC system consisting of an industrial camera, a 50 mm lens, and a constant cold light source was used. The camera was positioned approximately 500 mm from the specimen surface to minimize image distortion and ensure full coverage of the observation area. Prior to testing, a speckle pattern was prepared on the observation surface. A matte white base coating was first sprayed uniformly onto the specimen surface. After drying, random black speckles were applied using a marker pen, as illustrated in Figure 3b. During testing, image acquisition was synchronized with the loading system, with a capture frequency of one image every 400 ms. Load and displacement data were recorded simultaneously to establish correlations among load, deformation, and strain-field evolution.

To improve the quantitative interpretation of the DIC strain fields, a line-based analysis was performed for both compressive and splitting tensile specimens. For compressive specimens, a representative transect line approximately normal to the dominant high-strain localization band was selected from the peak-stage maximum principal strain contour, and the corresponding strain profile was extracted at different normalized stress levels. For splitting tensile specimens, three horizontal transect lines were arranged at y/H = 0.25, 0.50, and 0.75 to cross the vertical high-strain band along the loading centerline. Based on the extracted strain profiles, the peak strain on the line, the line-averaged strain, and the strain concentration factor were obtained to characterize the evolution of strain localization during loading. For splitting tensile specimens, the representative value at each stage was taken as the average of the three horizontal transect lines.

For splitting tensile specimens, the main crack position was identified from the maximum principal strain field. Virtual gauge-point pairs were arranged along the local normal direction of the crack at locations exhibiting maximum crack opening. The relative displacement between each point pair, compared with the initial state, was defined as the main crack opening displacement δ. To ensure comparability, identical selection criteria for gauge points were applied to all specimens, and the evolution of δ throughout loading was extracted accordingly.

#### 2.4.3. Scanning Electron Microscopy Analysis

SEM samples were collected from regions near the main crack after splitting tensile failure and cut into pieces approximately 10–20 mm in size. Prior to SEM observation, the samples were immersed in anhydrous ethanol for 24 h to reduce free pore solution and minimize continued hydration or geopolymerization during sample preparation and were subsequently dried below 50 °C until constant mass was achieved. Since an alkali-resistant basalt fiber was used in this study and no obvious fiber surface degradation related to this treatment was identified in the present observations, this step was considered not to cause significant interference with the fibers. Before SEM observation, the dried samples were sputter-coated with a thin layer of gold to improve surface conductivity and minimize charging during imaging. Microstructural observations were then conducted using a Thermo Fisher APRO high-resolution field-emission scanning electron microscope (Thermo Fisher Scientific, Waltham, MA, USA) to analyze the mechanisms by which basalt fibers influence the mechanical performance and crack evolution of the specimens.

#### 2.4.4. X-Ray Diffraction Analysis

Additional samples were taken from the central region of the splitting tensile specimens. After drying to constant mass, the samples were ground and sieved through a 200-mesh sieve. X-ray diffraction (XRD) analysis was performed using a Rigaku Ultima IV X-ray diffractometer (Rigaku Corporation, Akishima, Tokyo, Japan) with Cu Kα radiation, with a scanning range of 10–80°, scanning speed of 10°/min, and step size of 0.02°.

## 3. Results and Analysis

### 3.1. Analysis of Compressive Strength Results

**Figure 4:**
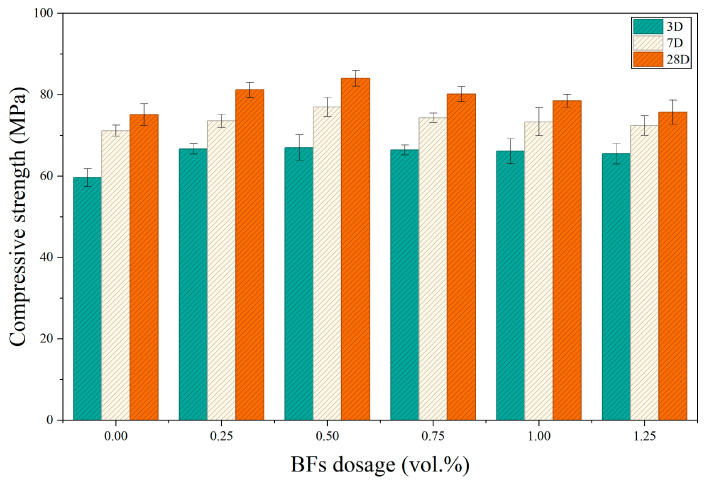
Effect of basalt fiber content on the compressive strength of BFCGGPC at different ages.

Figure 4 presents the compressive strength results of coal gangue–slag geopolymer concrete with different basalt fiber (BF) contents. Overall, the compressive strengths at 3, 7, and 28 days exhibited a trend of initially increasing and subsequently decreasing with increasing BF content, with the BF0.5 group showing the optimal performance. The compressive strengths of the control group (JZ) at 3, 7, and 28 days were 59.63, 71.17, and 75.10 MPa, respectively. Correspondingly, the BF0.5 group achieved strengths of 67.00, 77.00, and 84.05 MPa, representing increases of 12.36%, 8.20%, and 11.92% compared with the control group. Based on the 28-day results, the strength improvements relative to the JZ group were 8.12% (BF0.25), 11.92% (BF0.5), 6.72% (BF0.75), 4.53% (BF1), and 0.80% (BF1.25), indicating that an appropriate fiber dosage effectively enhances the compressive load-bearing capacity of the matrix.

The observed trend suggests that low to moderate BF contents suppress microcrack propagation through crack-bridging and stress-transfer mechanisms, thereby improving compressive performance. However, excessive fiber addition weakens the strengthening effect, likely due to reduced workability, poorer fiber dispersion, and increased internal defects [43,44,45,46,47]. Under the experimental conditions of this study, the optimal BF content for compressive strength is approximately 0.5%.

### 3.2. Analysis of Splitting Tensile Strength

**Figure 5:**
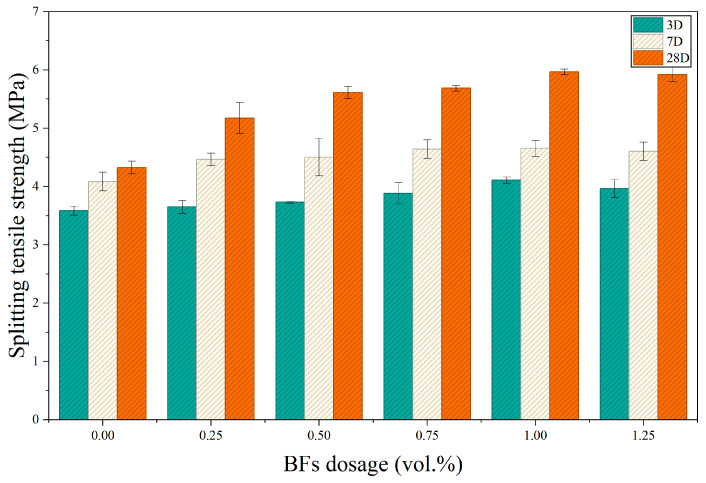
Effect of basalt fiber content on the splitting tensile strength of BFCGGPC at different ages.

Figure 5 shows the splitting tensile strength results for specimens containing different BF contents. Similar to compressive strength, splitting tensile strength at all curing ages first increased and then slightly decreased with increasing fiber dosage, indicating a pronounced reinforcing effect of basalt fiber on tensile behavior. At 3 days, the splitting tensile strength of the JZ group was 3.59 MPa, while the BF0.25, BF0.5, BF0.75, BF1, and BF1.25 groups reached 3.65, 3.73, 3.88, 4.11, and 3.97 MPa, respectively. At 7 days, strengths further increased, with the BF1 group achieving 4.70 MPa. At 28 days, the JZ group exhibited a strength of 4.33 MPa, whereas the BF0.25, BF0.5, BF0.75, BF1, and BF1.25 groups reached 5.14, 5.61, 5.69, 5.97, and 5.92 MPa, corresponding to increases of 18.7%, 29.6%, 31.4%, 37.9%, and 36.7%, respectively. The maximum value occurred in the BF1 group.

From the perspective of strength development, fiber-reinforced groups exhibited increases of 40.82–50.4% from 3 to 28 days, significantly higher than the control group. This indicates that basalt fiber improves both early-age crack resistance and medium-to-late-age tensile load capacity. The enhancement mechanism lies in the formation of an effective fiber-bridging network that inhibits microcrack initiation and propagation while dissipating fracture energy through fiber pull-out, interfacial debonding, and frictional sliding. When the fiber content increased to 1.25%, the strengthening effect plateaued and slightly declined, suggesting limited improvement under excessive fiber addition. Consequently, the optimal BF content for splitting tensile strength is approximately 1.0%.

### 3.3. Analysis of Flexural Strength

**Figure 6:**
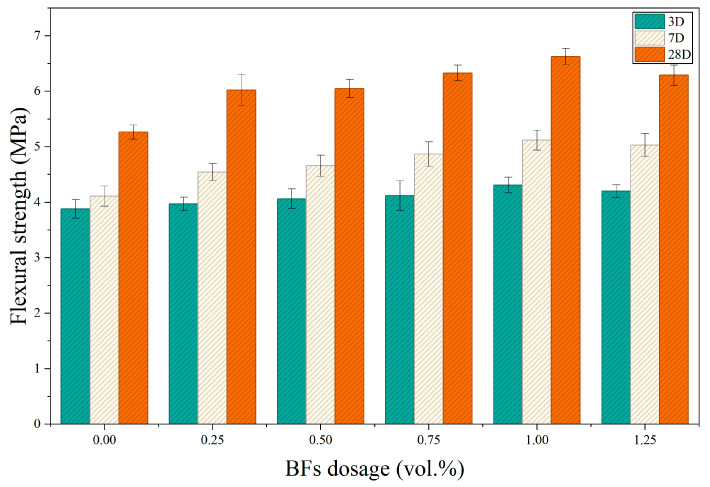
Effect of basalt fiber content on the flexural strength of BFCGGPC at different ages.

Figure 6 illustrates the flexural strength development of specimens containing different BF contents at 3, 7, and 28 days. Flexural strength increased continuously with curing age for all mixtures. At identical ages, strength first increased and then decreased with increasing BF content, with peak performance observed in the BF1 group.

The flexural strengths of the control group (JZ) were 3.88, 4.11, and 5.27 MPa at 3, 7, and 28 days, respectively. After fiber incorporation, flexural performance improved markedly, reaching maximum values at 1.0% BF content. The BF1 group achieved strengths of 4.31, 5.12, and 6.63 MPa at the respective ages, representing increases of 11.08%, 24.57%, and 25.81% compared with the control group. When the fiber content increased to 1.25%, flexural strengths slightly decreased to 4.20, 5.03, and 6.29 MPa but remained higher than those of the control group. These results indicate that basalt fiber effectively enhances flexural resistance within an optimal dosage range, while excessive fiber addition does not yield further improvement.

It should be noted that the optimal BF dosage depended on the loading mode. The compressive strength peaked at 0.5% BF, whereas the splitting tensile and flexural strengths reached their maximum values at 1.0% BF. This difference is attributed to the distinct failure mechanisms. Under compression, a moderate fiber dosage is sufficient to improve stress redistribution and reduce local strain concentration, whereas excessive fiber addition may reduce workability and increase internal defects. By contrast, tensile and flexural failures depend more strongly on crack bridging, interfacial debonding resistance, and pull-out energy dissipation, so a higher fiber dosage is required to form an effective crack-bridging network. Therefore, 0.5% BF is more suitable for compression-dominated performance, while 1.0% BF is more effective for tension- and flexure-dominated performance.

### 3.4. Analysis of Compressive Failure Evolution Based on DIC

**Figure 7:**
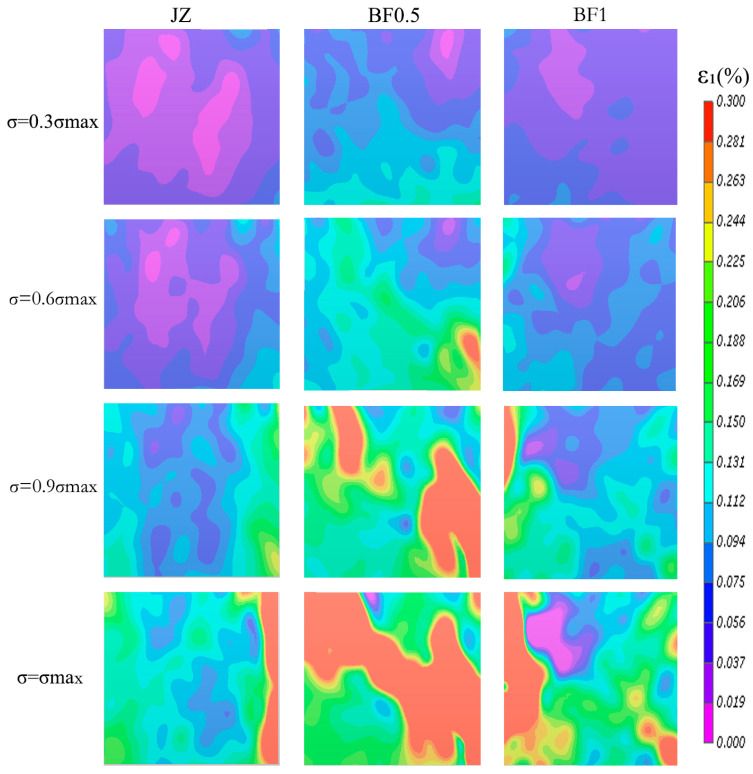
Evolution of surface strain fields during cube compression tests.

To clarify the influence of basalt fiber on compressive deformation and strain localization behavior, maximum principal strain contours of the JZ, BF0.5 (optimal compressive performance), and BF1 groups were compared at different normalized stress levels (Figure 7).

At σ = 0.3σ_max, strain distributions in all specimens were relatively uniform without continuous high-strain zones, indicating that deformation was dominated by pore compaction and near-elastic behavior. When stress increased to σ = 0.6σ_max, strain fields gradually evolved toward a nonuniform distribution. The JZ group still showed weak localization, whereas BF0.5 exhibited clearer localized strain regions, suggesting earlier stress redistribution induced by fiber bridging. The BF1 group displayed noticeable strain fluctuations as well.

At σ = 0.9σ_max, significant localization appeared in all groups but with distinct patterns. The JZ specimen showed a strong local concentration near the edges. In contrast, BF0.5 developed a broader and more continuous high-strain zone, indicating improved stress redistribution and reduced local concentration. Although BF1 also exhibited pronounced strain zones, their continuity was weaker than that of BF0.5. At peak stress (σ = σ_max), the JZ specimen displayed severe localized deformation leading to rapid failure, whereas BF0.5 maintained a wider strain distribution and better deformation compatibility. The BF1 group showed intermediate behavior.

To further quantify the strain localization characteristics described above, a line-based analysis was performed by extracting the maximum principal strain profiles from three horizontal transect lines, and the corresponding strain concentration factors were calculated, as summarized in Table 4. As shown in Table 4, clear differences in strain concentration were observed among the three compressive specimens. The BF0.5 specimen exhibited the lowest mean strain concentration factor, 3.356 ± 1.708, indicating that the increase in the maximum principal strain relative to the line-averaged strain was less pronounced and that the local strain peak was less sharp. The JZ specimen showed a mean strain concentration factor of 5.32 ± 1.147, indicating a noticeably stronger degree of local strain concentration than BF0.5. The BF1 specimen exhibited a mean strain concentration factor of 5.289 ± 4.080. Although its mean value was close to that of JZ, the much larger standard deviation indicates stronger localization differences among the three transect lines and poorer spatial uniformity of the internal strain distribution. Overall, the quantitative line-based indices indicate that BF0.5 showed the lowest degree of local strain concentration, whereas BF1 exhibited the largest spatial fluctuation.

These results demonstrate that basalt fiber primarily improves compressive performance by regulating strain localization evolution. Appropriate fiber content delays localization development through bridging and confinement effects, enhancing deformation coordination. Excessive fiber content weakens this optimization due to dispersion issues and increased defects. The BF0.5 group, therefore, exhibited the best compressive deformation compatibility, consistent with its highest compressive strength.

### 3.5. Analysis of Splitting Tensile Failure Evolution Based on DIC

#### 3.5.1. Evolution of Maximum Principal Strain

**Figure 8:**
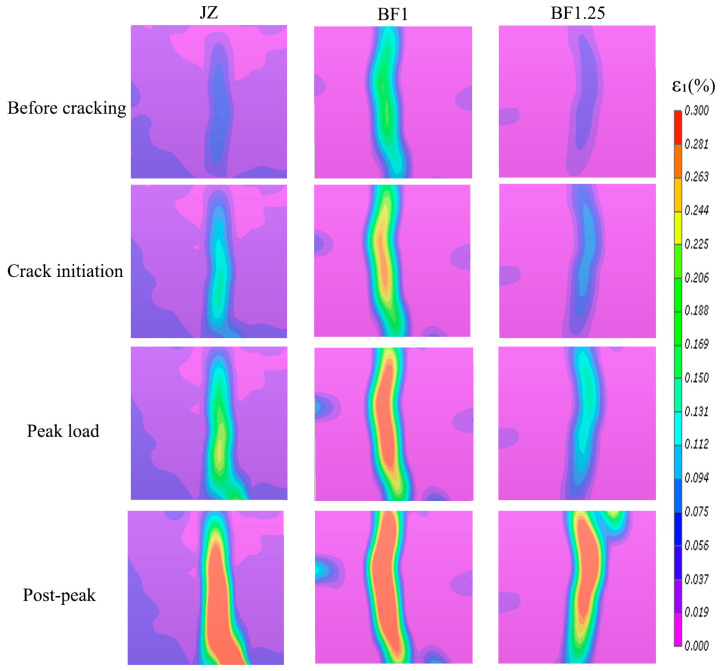
Maximum principal strain contour maps at typical stages during the splitting tensile process of specimens with different basalt fiber contents.

To clarify the influence of basalt fiber on the splitting tensile failure process of coal gangue–slag geopolymer concrete, the maximum principal strain contours of the control group (JZ), the specimen exhibiting the best splitting tensile performance (BF1), and the relatively high-fiber-content group (BF1.25) were selected for comparative analysis at four representative stages: pre-cracking, crack initiation, peak load, and post-peak, as shown in Figure 8. According to the previously described DIC methodology, the post-processed maximum principal strain field was used to characterize the evolution of surface local deformation. As illustrated in Figure 8, all specimens developed vertical high-strain zones near the loading centerline throughout the splitting tensile process, indicating that the main crack propagated along the tensile centerline, which is characteristic of typical splitting tensile failure.

Prior to crack formation, the JZ specimen exhibited only a weak and narrow localized high-strain region. In contrast, the BF1 specimen already showed a relatively continuous vertical strain concentration band, while the BF1.25 specimen demonstrated some improvement compared with the control group but with lower continuity and uniformity than BF1. These observations indicate that the incorporation of an appropriate amount of basalt fiber reduces premature deformation localization before macroscopic cracking, allowing the high-strain region to expand from a confined zone into a broader stress redistribution area.

During the crack initiation stage, strain localization in the JZ specimen rapidly intensified and was mainly concentrated in the middle–lower region, revealing the formation of a dominant single crack. The BF1 specimen developed a more continuous and through-height high-strain zone along the loading centerline, demonstrating that fiber bridging enhanced stress transfer and deformation coordination during crack initiation. Although the BF1.25 specimen also formed a continuous strain band, its concentration degree and continuity were weaker than those of BF1, suggesting that excessive fiber addition diminishes the regulation effect on early crack evolution.

At peak load, the JZ specimen showed pronounced strain localization concentrated along a single crack path. In comparison, the BF1 specimen formed the most continuous and extensive high-strain zone distributed along the tensile centerline, indicating that a larger volume of material participated in load bearing prior to reaching peak capacity, resulting in more stable crack propagation. The BF1.25 specimen also developed a distinct principal strain zone; however, its continuity and stability remained slightly inferior to those of BF1. This observation agrees well with the macroscopic splitting tensile strength results, where BF1 exhibited the highest strength, and BF1.25 showed a slight reduction.

In the post-peak stage, through-going cracks formed in all specimens, but their failure characteristics differed significantly. The JZ specimen exhibited severe strain localization concentrated along the main crack, followed by rapid, unstable failure typical of brittle materials. Conversely, the BF1 specimen maintained a wide and continuous vertical high-strain band after peak loading, indicating progressive crack propagation governed by energy dissipation mechanisms such as fiber bridging, pull-out, and interfacial debonding. The BF1.25 specimen displayed additional localized high-strain regions near the upper portion of the specimen, reflecting reduced strain-field uniformity under excessive fiber content, likely caused by fiber agglomeration or local defects inducing secondary localization.

As shown in Table 5, all the JZ, BF1, and BF1.25 specimens developed a pronounced strain localization band during splitting tensile loading, although their localization characteristics were different. The JZ specimen exhibited a mean strain concentration factor ${\bar{K}}_{L}$ of 5.206, showing a typical brittle localization response. The ${\bar{K}}_{L}$ value of BF1 increased to 6.761, indicating that a more pronounced high-strain core developed along the dominant crack path; however, combined with the crack opening displacement results in the following section, BF1 still showed a more stable and controlled localization evolution because rapid crack opening was effectively delayed. By comparison, the BF1.25 specimen exhibited an intermediate ${\bar{K}}_{L}$ value of 6.133, suggesting that further increasing the fiber content did not lead to additional improvement in crack regulation. Overall, the line-based quantitative results agree well with the maximum principal strain contours and the crack opening displacement analysis.

Overall, basalt fiber does not primarily reduce the magnitude of maximum principal strain but instead optimizes the strain localization pattern, enlarges the stress redistribution region, suppresses the rapid propagation of a dominant crack, and enhances post-peak energy dissipation capacity. Among all mixtures, the BF1 specimen exhibited the most favorable strain evolution characteristics and the most stable crack propagation behavior, indicating that an appropriate fiber dosage fully mobilizes the bridging and toughening effects of basalt fiber. When the fiber content increased to 1.25%, crack resistance remained superior to that of the control group, but no further improvement in toughening performance was observed. This conclusion is consistent with the splitting tensile strength results.

#### 3.5.2. Analysis of Main Crack Opening Displacement Evolution

**Figure 9:**
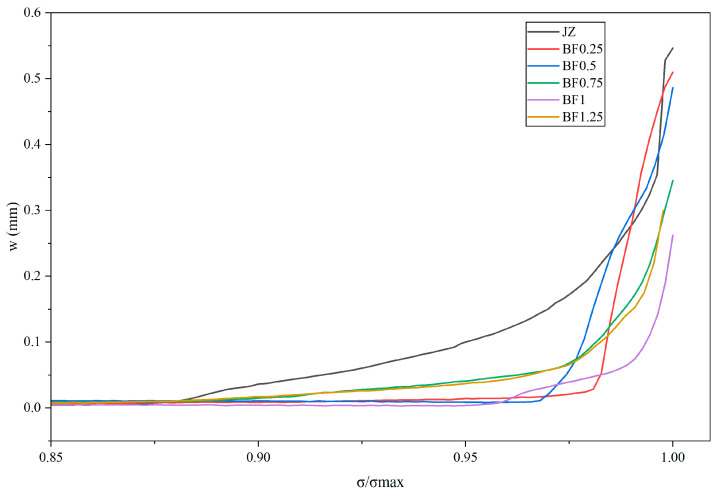
Evolution of main crack opening displacement under splitting tensile loading for specimens with different basalt fiber contents.

Figure 9 presents the relationship between the main crack opening displacement (δ) and normalized stress (σ/σ_max) for specimens with different basalt fiber contents. In general, crack-opening displacement increased progressively with loading; however, significant differences were observed in both the growth onset and the development rate among the specimens. The JZ specimen exhibited noticeable crack opening at relatively low normalized stress levels, followed by rapid growth near peak load, indicating that the fiber-free specimen entered unstable crack propagation at an earlier stage. After basalt fiber incorporation, crack opening development was clearly delayed, and peak crack opening displacement was significantly reduced, demonstrating the effectiveness of basalt fiber in restraining rapid crack opening. The BF1 specimen maintained the lowest crack-opening displacement throughout most of the loading process, showing the strongest crack-control capability. To quantitatively evaluate crack evolution characteristics, the rapid crack-opening initiation point (δ_0.9_) and the crack opening displacement at peak load (δ_u_) were extracted from the δ–σ curves, and the reduction rate of δ_u_ relative to the JZ group was calculated. The results are summarized in Table 6.

As shown in Table 6, basalt fiber significantly influenced crack opening behavior. Compared with the JZ specimen, all fiber-reinforced specimens exhibited higher δ_0.9_ values, indicating delayed transition from stable crack growth to rapid propagation. The BF1 specimen achieved the largest δ_0.9_ value (0.987), meaning that rapid crack development occurred at the latest stage. Meanwhile, the δ_u_ value of BF1 was only 0.262 mm, representing a 51.97% reduction compared with the control group, confirming its superior crack-opening inhibition near peak load. The BF0.75 and BF1.25 specimens also showed substantial reductions in δ_u_ (36.85% and 45.02%, respectively), demonstrating effective crack control but still slightly inferior to BF1. Lower fiber contents (BF0.25 and BF0.5) provided only limited reductions. Overall, an appropriate basalt fiber dosage effectively delays rapid crack opening and reduces peak crack width, with the BF1 mixture exhibiting optimal crack-control performance, consistent with its highest splitting tensile strength.

### 3.6. Micro-Mechanism Analysis

#### 3.6.1. SEM Micro-Mechanism Analysis

**Figure 10:**
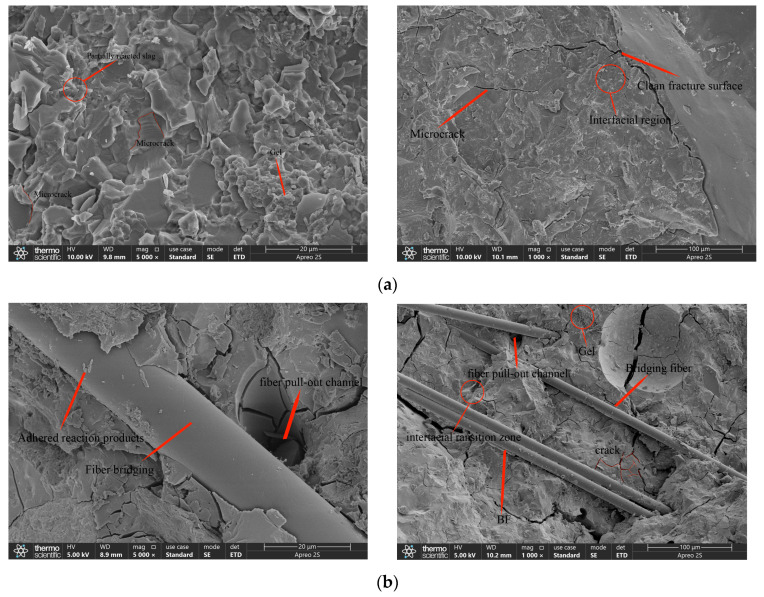
SEM images of typical fracture surfaces: (**a**) JZ; (**b**) BF1.

To further elucidate the strengthening mechanism of basalt fiber in coal gangue–slag geopolymer concrete, typical fracture surfaces were examined using SEM, as shown in Figure 10. The fiber-free specimen exhibited a typical brittle fracture morphology, characterized by interlaced flaky and blocky particles, accompanied by visible pores, microcracks, and discontinuities. These features indicate the presence of internal weak zones where cracks preferentially propagate along particle interfaces and structural defects during loading (Figure 10a).

After fiber incorporation, basalt fibers were observed bridging crack paths and firmly embedded within the matrix, confirming their crack-bridging function during fracture development. Reaction products adhered to fiber surfaces, indicating effective interfacial bonding between fiber and matrix. Localized fiber pull-out and interfacial debonding phenomena were also identified, demonstrating that crack propagation involved mechanisms such as fiber pull-out resistance, interfacial sliding, and frictional energy dissipation. These processes delay crack penetration and mitigate brittle failure behavior. It should be noted that an alkali-resistant basalt fiber was used in this study. Within the resolution of the present SEM observations, no clear signs of severe fiber surface corrosion or degradation were detected. Therefore, the slight decline in performance beyond the optimal fiber dosage is more reasonably attributed to poorer fiber dispersion, local agglomeration, and defect introduction at excessive BF contents, rather than to confirmed fiber degradation in the alkaline-activated matrix. Overall, the strengthening effect of basalt fiber arises primarily from crack bridging, interfacial load transfer, and pull-out energy dissipation, and its effectiveness depends strongly on fiber dispersion and interfacial integrity within the matrix.

#### 3.6.2. XRD Microstructural Analysis

**Figure 11:**
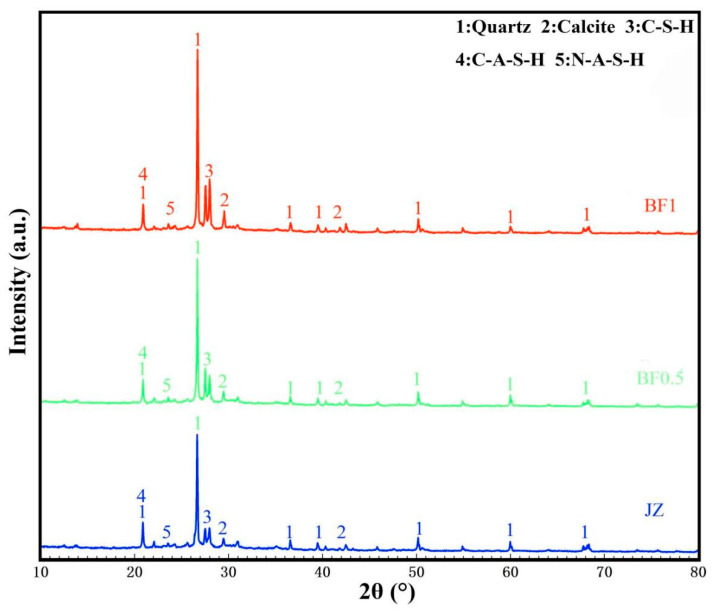
XRD patterns.

To investigate phase composition changes after basalt fiber incorporation, XRD analyses were performed on representative specimens (JZ, BF0.5, and BF1), as shown in Figure 11. The diffraction peak positions of all specimens were largely consistent, indicating that basalt fiber addition did not significantly alter the mineralogical composition of the geopolymer system. Distinct diffraction peaks located at approximately 20.8°, 26.6°, 36.5°, 39.5°, 50.1°, 59.9°, and 68.1° were mainly attributed to quartz. The strongest peak near 26.6° indicates the presence of residual crystalline SiO_2_ originating primarily from calcined coal gangue and quartz aggregates. A diffraction peak around 29.4° corresponds to calcite, likely formed through carbonation of calcium-containing reaction products during curing or testing.

A broad diffuse hump within the range of 20–35° indicates the presence of amorphous or poorly crystalline reaction products. Considering the compositional characteristics of the coal gangue–slag geopolymer system, this region mainly corresponds to C-(A)-S-H and N-A-S-H gels generated through the synergistic reaction between calcium-rich slag and aluminosilicate-rich coal gangue. These gel phases, together with residual crystalline materials, constitute the internal load-bearing skeleton governing mechanical performance.

In summary, quartz and minor calcite are the primary crystalline phases, accompanied by amorphous or low-crystallinity C-(A)-S-H/N-A-S-H gels. The incorporation of basalt fiber does not substantially alter the main phase assemblage detectable by XRD. However, subtle changes in the degree of geopolymerization cannot be completely excluded on the basis of XRD alone. Therefore, the performance enhancement observed in this study is interpreted mainly in terms of fiber bridging, interfacial stress transfer, and regulation of the fracture process.

## 4. Discussion

The present results are generally consistent with previous studies on basalt fiber-reinforced geopolymer materials, which have shown that basalt fiber is usually more effective in improving tensile, flexural, and fracture-related properties than compressive performance [22,26,35,36,43,44]. In the present study, the 28-day compressive strength reached its maximum at a BF content of 0.5%, whereas the splitting tensile strength and flexural strength achieved their peak values at 1.0%. In addition, the slight decrease in strength at higher BF contents is also in agreement with previous reports, in which excessive fiber addition reduced workability and fiber dispersion and promoted local agglomeration, thereby weakening the reinforcing effect [35,36,43,44].

A notable feature of the present results is that the optimal fiber dosage depends on the loading mode. Under compression, a moderate BF content was sufficient to improve the stress redistribution capacity of the matrix and reduce local strain concentration, resulting in the best performance at 0.5%. By contrast, under splitting tensile and flexural loading, a higher fiber content was required to form a more effective crack-bridging network and to enhance interfacial load transfer and pull-out energy dissipation so that the optimum content shifted to 1.0%. This difference indicates that basalt fiber plays a more significant role in crack control and post-cracking resistance than in directly improving compressive load-bearing capacity.

The DIC, SEM, and XRD results further support this interpretation. The DIC analysis showed that basalt fiber delayed strain localization and promoted more stable crack propagation. Under compression, the BF0.5 specimen exhibited a more uniform strain field and a lower degree of local strain concentration than the control group. Under splitting tension, the BF1 specimen showed a more stable strain localization band and a lower main crack opening displacement, indicating improved crack control during the full failure process. The SEM observations revealed typical fiber bridging, interfacial bonding, and pull-out features, confirming that the toughening effect mainly originated from crack bridging, interfacial load transfer, and fracture energy dissipation. Meanwhile, the XRD results showed that basalt fiber incorporation did not significantly alter the main phase composition of the geopolymer matrix. Therefore, the performance enhancement observed in this study is mainly attributed to physical toughening and interfacial mechanisms rather than substantial changes in the primary reaction products.

From an engineering perspective, the present results suggest that the selection of basalt fiber dosage in coal gangue-slag geopolymer concrete should be performance-oriented. When compressive performance is the primary objective, a moderate fiber dosage is more appropriate, whereas a relatively higher dosage is more beneficial when tensile behavior and crack resistance are of greater concern. At the same time, it should be noted that the present study mainly focuses on short-term mechanical behavior, crack evolution, and microstructural characteristics. The long-term durability and environmental competitiveness of basalt fiber-reinforced coal gangue-slag geopolymer concrete still require further investigation.

## 5. Conclusions

In this study, calcined coal gangue powder and slag were used as composite precursors to investigate the effects of varying basalt fiber (BF) contents on the mechanical properties, crack evolution behavior, and microstructural mechanisms of coal gangue–slag geopolymer concrete. Based on the experimental results, the following conclusions can be drawn:

(1) Basalt fiber effectively enhances the mechanical performance of coal gangue–slag geopolymer concrete; however, its strengthening effect shows clear content dependence. The 28-day compressive strength reached a maximum value of 84.05 MPa in the BF0.5 group, representing an 11.92% increase compared with the control group. The splitting tensile strength and flexural strength attained their peak values in the BF1 group, increasing by 37.88% and 25.81%, respectively, relative to the control specimen. These results indicate that basalt fiber provides a more pronounced improvement in tensile behavior and crack resistance than in compressive performance.
(2) DIC analysis demonstrates that basalt fiber modifies the evolution pattern of strain localization during both compressive and splitting tensile loading. Under compression, the BF0.5 group exhibited more favorable strain redistribution characteristics. Under splitting tension, the BF1 group showed a more stable crack propagation path and superior control of main crack opening displacement, indicating that the optimal fiber content varies with loading mode.
(3) SEM observations reveal that the strengthening and toughening mechanisms of basalt fiber primarily arise from crack bridging, interfacial load transfer, and energy dissipation associated with fiber pull-out. XRD analysis confirms that fiber incorporation does not significantly alter the primary phase composition of the geopolymer system. Therefore, performance enhancement is mainly attributed to regulation of crack propagation and interfacial interactions rather than changes in the dominant reaction products.
(4) Based on a comprehensive evaluation of macroscopic mechanical properties, DIC-derived crack evolution characteristics, and microstructural analysis, the optimal basalt fiber dosage within the scope of this study is 0.5% when compressive performance is the primary objective, and 1.0% when improving tensile behavior and crack resistance is the main objective.
(5) This study focuses on the short-term mechanical behavior, crack evolution, and microstructural mechanisms of BF-reinforced coal gangue–slag geopolymer concrete. No dedicated life-cycle assessment or long-term durability tests, such as sulfate resistance, carbonation, or freeze–thaw cycling, were conducted. Therefore, the environmental competitiveness and long-term service performance of this material system require further investigation.

## Figures and Tables

**Table 1:** Major chemical composition (mass%) and density of cementitious materials provided by the suppliers.

Materials	CaO	SiO_2_	Al_2_O_3_	SO_3_	Fe_2_O_3_	MgO	Balance *	Density
slag	35	33.5	17.5	1.65	1.03	6.01	5.31	3.12
coal gangue	0.15	53.5	42	-	1.8	-	2.55	2.8

* Balance was calculated by difference to 100% based on the supplier-provided major oxide composition and may include minor components and/or loss on ignition (LOI).

**Table 2:** Physical and mechanical properties of basalt fiber (BF) provided by the manufacturer.

Length (mm)	Fiber Density (g/cm^3^)	Tensile Strength (MPa)	Tensile Elastic Modulus (GPa)	Diameter/(μm)	Elongation at Break (%)
12	2.69	>2000	>85	17	2.5

**Table 3:** Mix proportions of BFCGGPC (kg/m^3^).

Test Group	CG	Slag	Water Glass	NaOH	Water	Sand	Coarse Aggregate	Fiber Dosage (vol.%)
JZ	239	239	194	34	73	618	1053	0
BF0.25	239	239	194	34	73	618	1053	0.25
BF0.5	239	239	194	34	73	618	1053	0.50
BF0.75	239	239	194	34	73	618	1053	0.75
BF1	239	239	194	34	73	618	1053	1
BF1.25	239	239	194	34	73	618	1053	1.25

**Table 4:** Line-based maximum principal strain indices for compression specimens.

Group	Line Position	$\varepsilon_{1,max}^{L}$	${\bar{\varepsilon }}_{1}^{L}$	$K_{L}$	${\bar{K}}_{L}$ ± SD
JZ	0.25	1.167	0.194	6.02	
	0.5	0.593	0.148	4	5.32 ± 1.147
	0.75	0.992	0.167	5.953	
BF0.5	0.25	0.821	0.38	2.158	
	0.5	1.246	0.48	2.598	3.356 ± 1.708
	0.75	2.765	0.521	5.312	
BF1	0.25	2.532	0.254	9.987	
	0.5	0.81	0.249	3.248	5.289 ± 4.080
	0.75	0.655	0.249	2.634	

Note: $\varepsilon_{1,\max}^{L}$ is the peak maximum principal strain on the transect line, ${\bar{\varepsilon }}_{1}^{L}$ is the line-averaged maximum principal strain, and the strain concentration factor is defined as $K_{L}=\varepsilon_{1,\max}^{L}/{\bar{\varepsilon }}_{1}^{L}$. The column “${\bar{K}}_{L}\pm SD$” represents the average value and spatial variation among the three horizontal transect lines within the same specimen, rather than the statistical variability among parallel specimens.

**Table 5:** Line-based maximum principal strain indices for splitting tensile specimens.

Group	Line Position	$\varepsilon_{1,max}^{L}$	${\bar{\varepsilon }}_{1}^{L}$	$K_{L}$	${\bar{K}}_{L}$ ± SD
JZ	0.25	2.604	0.491	5.299	
	0.5	4.292	0.785	5.471	5.206 ± 0.321
	0.75	4.712	0.972	4.848	
BF1	0.25	4.101	0.578	7.097	
	0.5	4.439	0.746	5.949	6.761 ± 0.707
	0.75	4.049	0.559	7.236	
BF1.25	0.25	3.754	0.648	5.792	
	0.5	3.669	0.564	6.502	6.133 ± 0.356
	0.75	2.683	0.439	6.106	

Note: $\varepsilon_{1,\max}^{L}$ is the peak maximum principal strain on the transect line, ${\bar{\varepsilon }}_{1}^{L}$ is the line-averaged maximum principal strain, and the strain concentration factor is defined as $K_{L}=\varepsilon_{1,\max}^{L}/{\bar{\varepsilon }}_{1}^{L}$. The column “${\bar{K}}_{L}\pm SD$” represents the average value and spatial variation among the three horizontal transect lines within the same specimen, rather than the statistical variability among parallel specimens.

**Table 6:** Characteristic parameters of main crack opening evolution.

Group	Fiber Content (%)	$\lambda_{w}$	$w_{p}$ (mm)	$\mathrm{Reduction}\;\mathrm{Rate}\;\mathrm{of}\;w_{p}$ Relative to JZ (%)
JZ	0	0.958	0.547	0.00
BF0.25	0.25	0.985	0.510	6.69
BF0.5	0.50	0.979	0.487	10.97
BF0.75	0.75	0.978	0.345	36.85
BF1	1.00	0.987	0.262	51.97
BF1.25	1.25	0.977	0.300	45.02

Note: $\delta_{0.9}$ represents the normalized stress corresponding to the transition from stable to rapid crack opening; $\delta_{u}$ denotes the crack opening displacement at peak load. The reduction rate relative to the JZ group is calculated as: $R=\frac{\delta_{u}^{\mathrm{JZ}}-\delta_{u}^{i}}{\delta_{u}^{\mathrm{JZ}}}\times 100\%$, where $\delta_{u}^{\mathrm{JZ}}$ is the peak crack opening displacement of the control group and $\delta_{u}^{i}$ corresponds to that of the *i*-th specimen group.

## Data Availability

The original contributions presented in this study are included in the article. Further inquiries can be directed to the corresponding authors.
